# Late Presentation of Brachial Plexus Traction Injury After Fixation of Chronic Clavicle Nonunion Mimicking Brachial Plexitis: A Case Report and Literature Review

**DOI:** 10.7759/cureus.102930

**Published:** 2026-02-03

**Authors:** John G Skedros, John T Cronin, Kevin B Curtis, Jessie A Montgomery, Brett W Richards, Mark A Mahan

**Affiliations:** 1 Shoulder and Elbow, Utah Orthopaedic Specialists, Salt Lake City, USA; 2 Medical School, Spencer Fox Eccles School of Medicine, University of Utah, Salt Lake City, USA; 3 College of Life Sciences, Brigham Young University, Provo, USA; 4 Neurosurgery, University of Utah, Salt Lake City, USA

**Keywords:** brachial plexus injury, clavicle fracture nonunion, clavicle reconstruction, double hit phenomenon, modified oberlin nerve transfer, parsonage turner syndrome

## Abstract

We report the case of a healthy 56-year-old male who developed severe neuromotor deficits of his upper extremity after two surgeries for a chronic clavicle fracture nonunion. Initial reconstruction with plate fixation achieved anatomic alignment. Twelve days later, diffuse numbness and weakness developed in his ipsilateral hand. A second (and final) clavicle surgery was then performed to increase the subclavicular space, after which his neuromotor deficits dramatically worsened. Because brachial plexopathy, specifically Parsonage-Turner syndrome (PTS), was considered the most likely diagnosis, no further clavicle or brachial plexus surgery was performed, as this would likely not alter the course of this inflammatory condition. Retrospective analysis suggests that iatrogenic nerve traction injury was the most likely cause of the deficits, rather than brachial plexopathy/PTS. The initial 12-day delay in symptom onset was perplexing and created diagnostic uncertainty. We report this intriguing case to: (1) discuss this diagnostic dilemma in the context of a literature review of the two competing diagnoses and the surgical and non-surgical decisions that were made, and (2) provide details of the patient’s recovery over seven years. These findings offer valuable considerations and clinical data for clinicians and surgeons tasked with managing patients with significant neuromotor complications after clavicle fracture surgery.

## Introduction

Neurovascular complications are of particular concern with clavicle fracture fixation/reconstruction surgeries due to the close proximity of the clavicle to the brachial plexus and subclavian vessels. Reported complications include brachial plexopathy, thoracic outlet syndrome (TOS), pseudoaneurysm, arteriovenous fistulas, and deep vein thrombosis [1]. Prior literature has suggested that the frequency and etiology of these potential complications may be influenced by the time between injury and initial surgery [1]. This is particularly important in cases of nonunion or malunion, where abundant fracture callus or altered clavicle length may have compressive or traction/stretch effects.

This report addresses the diagnostic uncertainty between neurogenic TOS, a well-known complication of both operative and non-operative treatment of clavicle fractures [1-11], and Parsonage-Turner syndrome (PTS), a brachial plexopathy characterized as an inflammatory/immune-mediated condition [12-15]. PTS is typically heralded by the sudden onset of severe shoulder pain followed by atrophy of the muscles innervated by the affected nerves with corresponding sensory deficits [13,16-18]. Difficulty with finger flexion, shoulder abduction and external rotation, forearm pronation, and winging of the scapula are common deficits [13,19]. In addition to being called acute brachial plexitis/plexopathy and PTS, this condition is known by many other names, including neuralgic amyotrophy, acute brachial neuropathy, brachial plexus neuropathy, idiopathic brachial neuritis/plexopathy, paralytic brachial neuritis, brachial radiculitis, localized/multiple neuritis of the shoulder girdle, and others [13,15,20]. The condition was described as early as 1887 and was further characterized in 1948 by Parsonage MJ and Turner JW's seminal study of 136 patients [13,16]. The diagnosis of PTS is often a “diagnosis of exclusion,” where nothing else fits the constellation of signs and symptoms [21,22]. Because PTS is presumed to be inflammatory in nature, surgical intervention is generally not expected to alter its course [13,23].

We report a complex case of neuromotor deficits developing after surgical reconstruction of a clavicle fracture nonunion in detail in order to: (1) alert surgeons that both iatrogenic neurapraxia and brachial plexopathy/PTS can manifest with delayed symptoms (several days to 1-2 weeks) after surgical reconstruction of a chronic midshaft clavicle fracture nonunion and can become severe (i.e., progress to axonotmesis); (2) discuss the viability of the competing diagnoses that were considered (i.e., PTS vs. the more plausible occurrence of iatrogenic traction injury to the brachial plexus); (3) suggest possible preventative measures that should be considered at the time of surgery, particularly for avoiding atypical compression or neuropraxic etiologies; (4) emphasize the importance of having a surgeon available who is capable of performing brachial plexus dissection/neurolysis if needed; and (5) in the context of a literature review, provide a detailed longitudinal account of one patient’s functional recovery over a seven-year period and potential outcomes if not addressed with appropriate revision surgery.

## Case presentation

**Figure 1:**
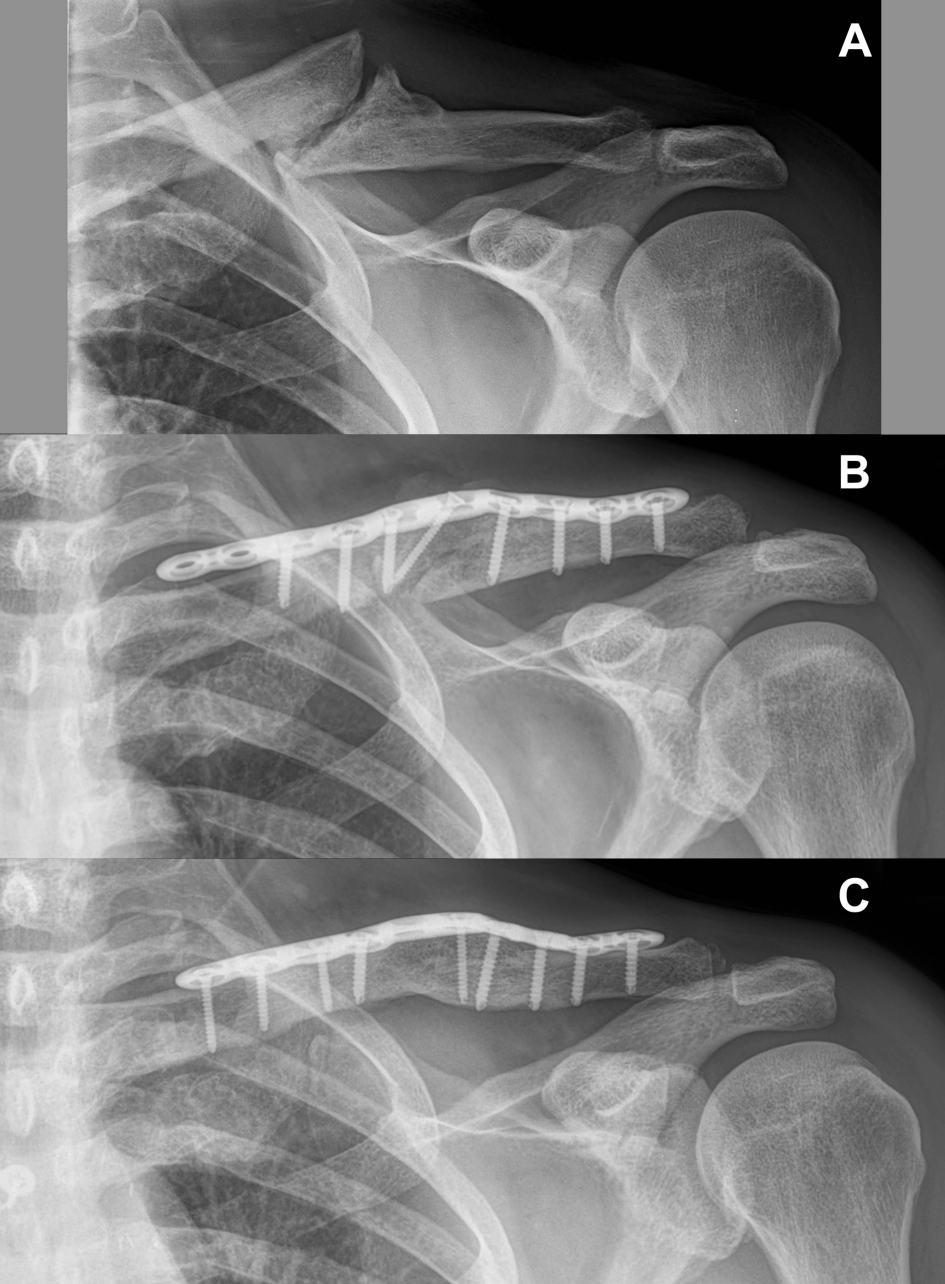
Anterior-to-posterior radiographs of the patient’s left shoulder. These radiographs were taken: (A) in September 2018, two months before the patient’s index clavicle surgery, and show a hypertrophic midshaft clavicle fracture nonunion; (B) in early November 2018, and show the hardware after the index clavicle reconstruction surgery, which resulted in some clavicle lengthening; and (C) in 2024, and show the state of the hardware after the revision surgery (performed in mid-November 2018), when additional clavicle lengthening occurred because the fracture was gapped open further and the metal plate was bowed upward. The clavicle fracture is well healed.

A 56-year-old right-hand-dominant healthy male (83.5 kg, 180.3 cm, BMI 25.7) was seen in our clinic for a chronic nonunion left-sided midshaft clavicle fracture that occurred 15 years prior due to a fall while running. This was a closed fracture, and no surgery had been done (Figure 1A). The patient was employed as a vehicle fleet technician for a trucking company, which often required intensive manual labor (e.g., lifting 36 kg brake drums and 54 kg wheels). Because of worsening pain at the fracture site during increased work-related lifting activities, the patient requested surgery to address this problem. He had no prior history or evidence of neurovascular or motor deficits. He had type 2 diabetes (with a normal hemoglobin A1c, 5.6%) and hypertension, which were treated with metformin and losartan. He did not drink alcohol or smoke. He had been in a motorcycle accident 23 years prior that resulted in a concussion that recovered fully within one month; there had been no work-up, imaging, or hospitalization. As discussed below, this prior incident was potentially relevant at the time that his brachial plexopathy occurred.

The first clavicle surgery

In early November 2018, bone grafting and plating of the clavicle fracture was performed without any apparent complications and without a perioperative nerve block (Figure 1B). The fracture surfaces and medullary canal orifices at the fracture site were abraded of fibrous tissue and burred to create punctate bleeding. Demineralized allograft human bone matrix in gel (Grafton^TM^ Gel, Medtronic, Minneapolis, Minnesota, USA) and the hypertrophic osteophytes at the fracture site were morselized and used to graft within the fracture gap and into the adjacent medullary canals. Bone graft was not placed around the fracture site [24].

Anatomic reduction was achieved with placement of screws and a metal plate on the dorsal clavicle surface (Figure 1B). The orthopaedic surgeon did not believe any significant compression of the subclavicular structures had occurred because seemingly little effort was required to satisfactorily align the clavicle, and a 0.25 inch (6.4 mm) periosteal elevator could be slid beneath and posterior to the reconstructed fracture site. Release of tissue farther medial and lateral from the fracture site [24,25] was avoided to minimize disruption of blood flow [26]. There were no apparent complications.

The second (and final) clavicle surgery

At 12 days after surgery, the patient noted diffuse numbness in his ipsilateral hand and progressive weakness (3/5) in wrist extension, finger flexion, and grip strength. Physical examination revealed no vascular deficits and no hand edema, obvious hematoma, or increased swelling near the clavicle or in the arm or forearm. There were no changes in his voice or respirations, and no increased pain (brachial plexopathy/PTS typically presents with high pain) [13,17]. The surgeon believed that iatrogenic TOS from the anatomic re-alignment of the clavicle was the most likely diagnosis, but the delayed onset of symptoms was puzzling.

Due to his prior firsthand experience with an apparently similar case [2], the surgeon recommended prompt surgical intervention. In mid-November 2018 (16 days after the index clavicle surgery), a second surgery was done to decompress the subclavicular space by gapping open the fracture site by 12 millimeters, including bending the center of the metal plate upward (Figure 1C). During this revision surgery, a burr was also used to remove approximately three millimeters of bone from the inferior surface of the middle one-third of the clavicle. These steps were similar to those that the surgeon had taken in order to successfully decompress the subclavicular space of his prior patient with iatrogenic TOS after open reduction and internal fixation (ORIF) for a clavicle fracture nonunion [2], and as subsequently described by others for similar circumstances [27-29]. There seemed to be ample space inferior and posterior to the clavicle, but the subclavicular tissue was somewhat sclerotic. No attempt was made to dissect through this sclerotic tissue or to release adherent tissue along a larger portion of the clavicle because of the risk of subclavian vessel injury [30,31], and because the orthopaedic surgeon did not have a neurosurgeon available who could perform neurolysis of the brachial plexus at that time.

Marked worsening of symptoms immediately after second clavicle surgery

Immediately after the second and final clavicle surgery, the patient exhibited a marked worsening of sensory and motor functions, including new-onset complete wrist drop. Upon examination, testing of left upper extremity muscles revealed the following: deltoid 4+/5, biceps 2/5, triceps 3 to 4-/5, wrist extension 0/5, extension of the digits 0/5, thumb flexion 3+/5, and grip strength 3/5. There was decreased perception of light touch over the first, second, and third digits, and, to a lesser extent, over the fourth and fifth digits. Video 1 shows the patient’s active left-hand motions obtained eight days after the revision surgery (he is wearing a cock-up wrist splint for complete wrist drop).

**Video 1:** November 2018 follow-up visit.

These findings were consistent with brachial plexopathy, rather than the more typical TOS that can occur after the surgical fixation of acute, sub-acute, and nonunion clavicle fractures (as reviewed and compared by Clitherow HD and Bain GI [10] and Borole A et al. [1]). The sudden worsening of symptoms following a 12-day delay in their onset created uncertainty as to what should be done next.

Work-up within 14 days of the second/final clavicle surgery

**Figure 2:**
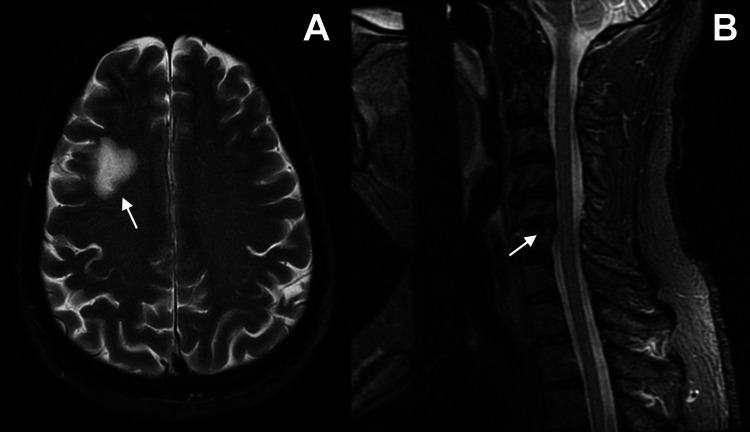
MRI of the patient’s head and neck. MR images of our patient’s head and neck obtained after the second clavicle surgery. (A) Axial T2 image showing a right frontal lobe lesion, likely related to a prior motorcycle accident, and (B) sagittal STIR image showing moderate degenerative disc disease at the C5-C6 level, causing moderate bilateral foraminal stenosis and mild central canal stenosis. As discussed below, it was initially hypothesized that these lesions might have contributed to a double- or triple-crush syndrome leading to the patient’s neurological deficits after the revision/final clavicle surgery. STIR: Short Tau Inversion Recovery.

The patient was started on a three-day course of IV methylprednisolone followed by a ten-day oral prednisone taper [32-34]. He remained in the hospital for three days for additional work-up and evaluation, which included advanced imaging studies and consultations from a neurologist and a neurosurgeon. MRI of his brain and neck identified two lesions: (1) a right frontal lobe lesion (Figure 2A), and (2) moderate degenerative disc disease at cervical intervertebral level C5-C6 causing moderate bilateral foraminal stenosis and mild central canal stenosis (Figure 2B). A CT angiogram of his head revealed a two-centimeter region of diminished density in the right periventricular frontal lobe that extended to the cortical gray matter; this corresponded to the lesion seen in the MRI in Figure 2A. There was no mass effect or hemorrhage, no midline shift, and no hemodynamic stenosis or evidence of stroke. The finding in the right frontal lobe was likely from his remote motorcycle accident. These abnormalities in the head and neck were not considered significant factors in his left upper extremity symptoms.

**Figure 3:**
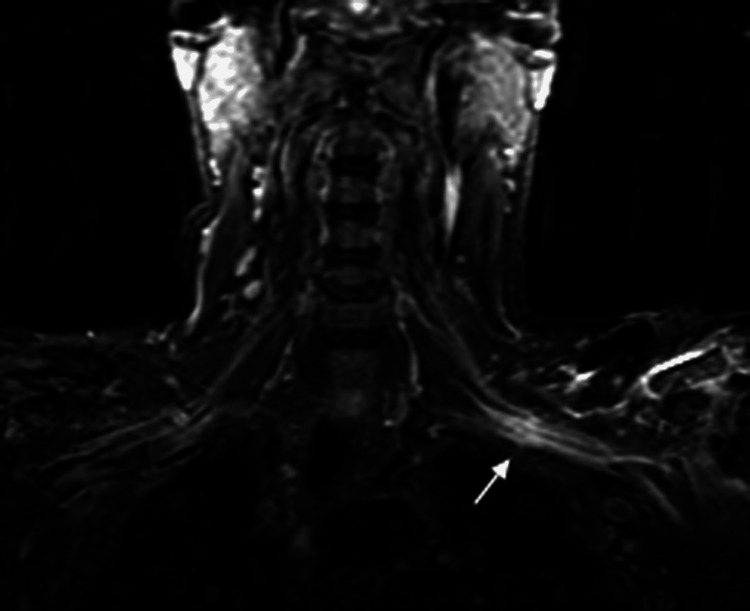
Coronal T2-STIR MR image of the patient’s left shoulder. The arrow shows enhancement of the left brachial plexus. Because of this diffuse signal abnormality, the neurosurgeon consultants opined that the patient’s deficits were predominantly inflammatory in nature and consistent with PTS. Therefore, additional surgery was not recommended, as it was believed that further surgical manipulation or dissection could worsen the patient’s deficits. PTS: Parsonage-Turner syndrome; T2-STIR: T2-weighted Short Tau Inversion Recovery.

Although MRI of the left brachial plexus region did not reveal evidence of any space-occupying lesion that might significantly compress this region [1], there was soft tissue swelling and induration associated with the clavicle that was mildly impinging on the neurovascular bundle at the mid-clavicle (Figure 3). In view of this finding, the patient's surgeon then considered performing hardware removal with excision of a portion of the clavicle and asked a neurosurgeon consultant to participate by performing neurolysis of the brachial plexus in the affected region. It was speculated that a double- or triple-crush phenomenon from the proximal neurologic lesions was a contributing factor [2,35,36] (as discussed below, an alternative explanation described as a “double-hit phenomenon” by Gross CE et al. was not known to the surgeon at that time) [37]. However, the neurosurgeon consultant recommended against immediate revision surgery because of the perplexing finding that the entire brachial plexus showed high signal intensity on T2-STIR images (Figure 3) [38]. His opinion was that an inflammatory process (e.g., PTS) was at play and explained the 12-day delay in symptom onset [39]. Thus, he believed that surgical intervention might precipitate additional deficits. Milner CS et al. [18] provide support for this, reporting that 14 of their 38 patients had a delay of 1-14 days in the manifestation of brachial plexopathy/PTS in association with antecedent surgery. Notably, none of the surgeries in their patients were done in the clavicle area.

The patient was discharged from the hospital with the plan for periodic re-evaluations over the next 1-2 weeks. Evaluation by a vascular surgeon reaffirmed the opinion that he did not have vascular TOS and, therefore, hardware removal with midshaft clavicle resection and/or first rib resection was deemed unnecessary. During that time, the patient had progressively worsening “burning pain” diffusely in his ipsilateral (left) hand. It was clear that the patient needed more advanced brachial plexus care.

Referral to a neurosurgeon with expertise in brachial plexus injuries

The patient was then sent for an evaluation by a neurosurgeon with advanced experience in managing brachial plexus injuries. This neurosurgeon first saw the patient 18 days after the final clavicle fracture surgery and continued to see him regularly in his clinic over the next three years. At the initial consultation, the neurosurgeon believed the patient had a relatively classic onset of neuropathic pain and deficits after a physiological stressor, with delayed-onset weakness and atrophy of the left upper extremity. The differential diagnosis at that time included amyotrophic neuralgia (i.e., brachial plexopathy/PTS), cervical radiculopathy, brachial plexus traumatic injury, mononeuritis multiplex, and/or diabetic amyotrophy.

The first electrodiagnostic study

At 26 days after the second/final clavicle-area surgery, a nerve conduction study (NCS) with electromyography (EMG) analysis was done and showed substantial deficits involving all trunks of the brachial plexus (mainly the middle and lower trunks, with sparing of the deltoid/axillary nerve). The lack of voluntary contractions of some muscles and prominent fibrillation potentials were suggestive of brachial plexopathy/PTS; however, trauma or traction/stretch etiologies that cause axonotmesis could not be excluded [13,40]. Given these findings, along with the MRI findings in Figure 3, the diagnosis of brachial plexopathy/PTS was again considered most likely (both neurosurgeon consultants drew this conclusion). Non-operative management was therefore continued, and the patient was informed that sensation and strength in his arm would slowly improve over the course of 2-3 years, with the possibility of residual deficits, as is typical of brachial plexopathy/PTS [18,40-42]. Gabapentin was prescribed for neuropathic pain [25,43].

Selective cervical nerve root injection

Four months after the final clavicle surgery (March 2019), the patient had a selective corticosteroid injection of his left C6 nerve root to assess whether nerve root compression was contributing to his persistent symptoms [44]. This provided no relief, which is not surprising considering that over 50% of patients with brachial plexopathy/PTS show abnormalities on cervical spine MRI or CT that do not correlate with their clinical presentation or peripheral nerve findings [13,17,45].

Follow-up at 4-6 months and the second (and final) electrodiagnostic study

Over the next four months, the patient exhibited very slow improvements in some functions, including modest recovery in finger and wrist extension. However, he still had profound deficits in elbow flexion. A video was also obtained of his hand motions at 5.5 months (May 2019) after the final clavicle surgery (Video 2).

**Video 2:** May 2019 follow-up visit.

At that time (May 2019), the patient underwent surgery to transfer fascicles from the median nerve supplying the flexor carpi radialis muscle to the biceps muscle, known as a modified Oberlin nerve transfer procedure [46,47]. Six weeks later, the patient demonstrated significant improvement in elbow flexion strength. However, his left-hand atrophy and weakness persisted.

A repeat electrodiagnostic study showed evidence of a diffuse, patchy brachial plexopathy involving most of the plexus. Fibrillation potentials were present in all muscles studied except the deltoid. Sensory nerve conduction studies showed non-recordable median, ulnar, radial, and lateral antebrachial cutaneous responses, while the medial antebrachial cutaneous remained present. Motor evaluation revealed prolonged latency of the median nerve at the wrist and decreased conduction velocities of the ulnar nerve at the elbow. Electromyographic analysis revealed profound insertional activity throughout the left upper extremity, including the biceps, triceps, first dorsal interosseous, abductor pollicis, extensor digitorum communis, flexor digitorum superficialis, and flexor carpi ulnaris muscles.

Ultrasound-guided evaluations revealed enlargement of the median nerve at the proximal carpal tunnel and decreased echogenicity of the ulnar nerve in the ulnar groove at the elbow. In situ cubital tunnel and carpal tunnel releases were then performed to eliminate any local compressive factors that could impede nerve recovery. Six weeks later, mild improvements in grip strength and key pinch were noted. For several months after these procedures, he had biweekly sessions with a hand therapist [48,49].

At five months after the peripheral nerve decompression surgeries, the patient showed increased grip strength, but notable atrophy of the first dorsal interosseous muscle and poor dexterity persisted. His diffuse hand numbness, though still present, was improving. The patient had returned to work for the trucking company, but in the capacity of a supervisor that did not require manual labor.

Follow-up 27 months after final clavicle surgery

**Figure 4:**
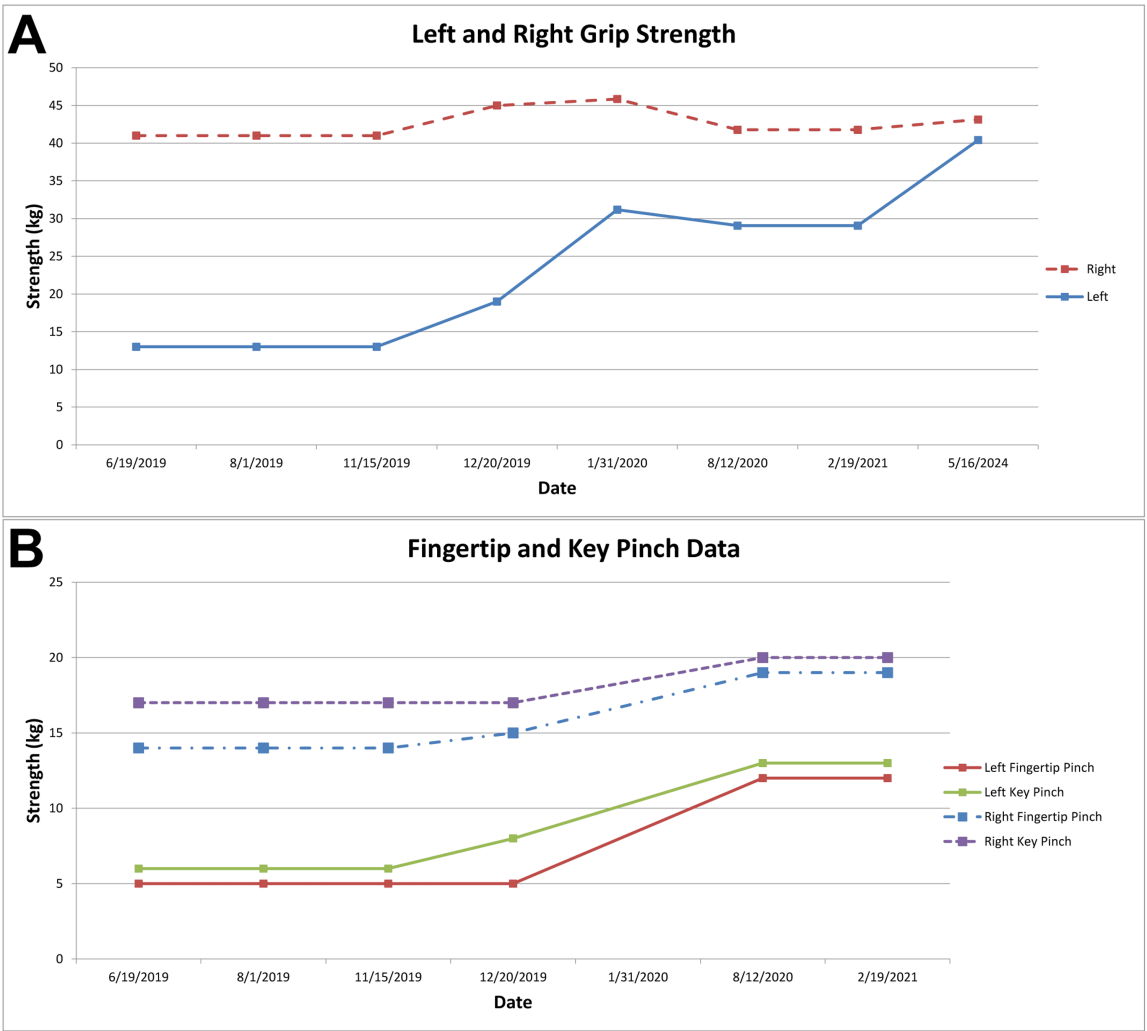
Grip strength, fingertip pinch strength, and key pinch strength. (A) Grip strength data from June 2019 to November 2021. By November 2025, the patient’s left-hand grip strength and the left-versus-right grip strength difference remained within 5% of the November 2021 values. Improvement is reflected by increasing values in the involved (left) hand. (B) Fingertip pinch and key pinch strength data from June 2019 to February 2021 (these data were not obtained after February 2021). Improvement is reflected by increasing values in the involved (left) hand.

At a follow-up with the neurosurgeon specialist in February 2021 (27 months after the final clavicle surgery), the patient demonstrated impressive recovery of elbow flexion and grip strength (Figure 4), but numbness and neuropathic pain persisted in his hand. A video was also obtained of his hand motions at 36 months (mid-November 2021) after the final clavicle surgery (Video 3).

**Video 3:** November 2021 follow-up visit.

A case similar to our patient is “discovered”

In November 2021, we encountered a case report by Gross CE et al. [37] describing a patient with postoperative symptoms similar to those of our patient. However, their patient was not considered to have brachial plexopathy/PTS. Notably, they described their case as “previously unreported.” Their patient was a 78-year-old female who developed symptoms of severe brachial plexopathy 24 hours after surgical fixation of a comminuted midshaft clavicle fracture, which was done five weeks after the injury. As in our case, they initially performed restoration of anatomical alignment of the clavicle using standard ORIF with a dorsally placed metal plate and screws. Notably, at the time of their index surgery, the clavicle was lengthened by 27 millimeters (mm) after five weeks. Due to the acute brachial plexopathy, at approximately 36 hours after their index clavicle fixation surgery (12 hours after the onset of symptoms), they removed the hardware and allowed the clavicle to shorten to the pre-operative length (in our revision surgery we lengthened the clavicle). They did not perform brachial plexus neurolysis. Their patient then exhibited immediate improvement, and an excellent outcome was achieved by eight months.

Gross CE et al. [37] reported the overall length of their patient’s clavicle both before and after surgery. We retrospectively determined that our index fixation increased our patient’s clavicle length by approximately 12 mm. After our revision surgery, the patient’s clavicle length increased by an additional 13 mm (a total increase of 25 mm, compared to 27 mm reported by Gross CE et al.). Hence, in retrospect, perhaps the most appropriate intervention in our patient’s case would have been to remove the metal plate and screws and allow the clavicle to shorten back to its baseline (pre-operative) length. However, knowledge of this intervention was not known to any of the providers involved in the decision-making/management of our patient’s perioperative complication.

**Figure 5:**
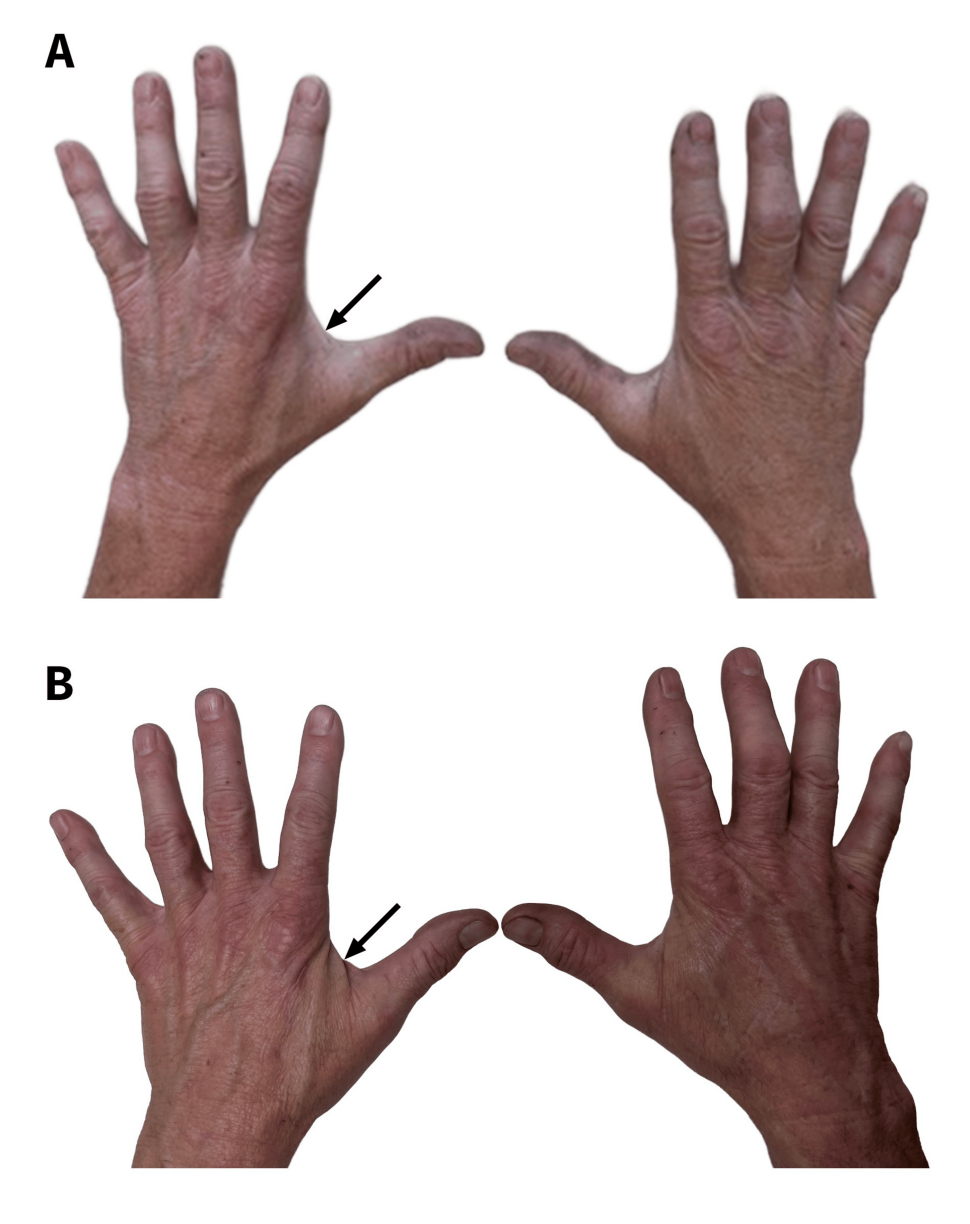
Photographs of the patient’s hands. The photographs were taken in February 2024 (A) and November 2025 (B). The arrows point to mild atrophy of the first dorsal interosseous muscle of the patient’s left hand, which is ipsilateral to the side of the clavicle fracture surgeries. Direct physical examination showed that the atrophy was similar in (A) and (B). This atrophy correlates with the persistent weakness in the patient’s left hand at his final (seven-year) follow-up.

Fortunately, our patient continued to improve, and his clavicle fracture healed well and became asymptomatic by four months after the final surgery. In February 2024 (63 months after the final clavicle surgery), a follow-up radiograph was obtained (Figure 1C). At 63- and 84-month follow-ups, photographs of his hands were obtained (Figure 5), which showed similar atrophy of the first dorsal interosseous space. At that 63-month follow-up, he reported normal sensation and function of his shoulder girdle, shoulder, arm, and forearm. His left-hand tripod pinch was 17% less than his right hand (Figure 4).

Grip strengths were compared between his 63- and 84-month (November 2025) follow-ups. His overall grip strength had plateaued at that 63-month follow-up, with left-hand grip strength being approximately 93.5% of that in his contralateral (dominant) hand (40.4 vs. 43.1 kg, respectively). The normal difference between a non-dominant and dominant hand is about 10% [50,51].

At his 63-month follow-up, he reported trace numbness in his fingertips. Two-point discrimination was impaired in his thumb, ring finger, and long finger (radial aspect), measuring 2 mm greater than the opposite (normal) hand. The fingertips of the normal hand were all less than the 6 mm normal threshold [52]. The small finger and ring fingertips (ulnar aspect) showed greater deficits; namely, 6 mm greater than that of the two-point discrimination on the same locations of the normal hand.

By his 84-month (seven-year) follow-up, two-point discrimination was nearly normal for fingertips in the median and radial nerve fields (within 1.0 mm of the opposite hand), but was 9 mm for fingertips in the ulnar nerve field (vs. 4.0 mm for the normal hand ulnar nerve field). At this visit, he reported occasional cramping of the left hand over the preceding 1.5 years during activities such as typing and grasping. He stated that the reduced use of his left hand “minimized this nuisance.”

Additional outcome data recorded over the seven-year follow-up period

**Figure 6:**
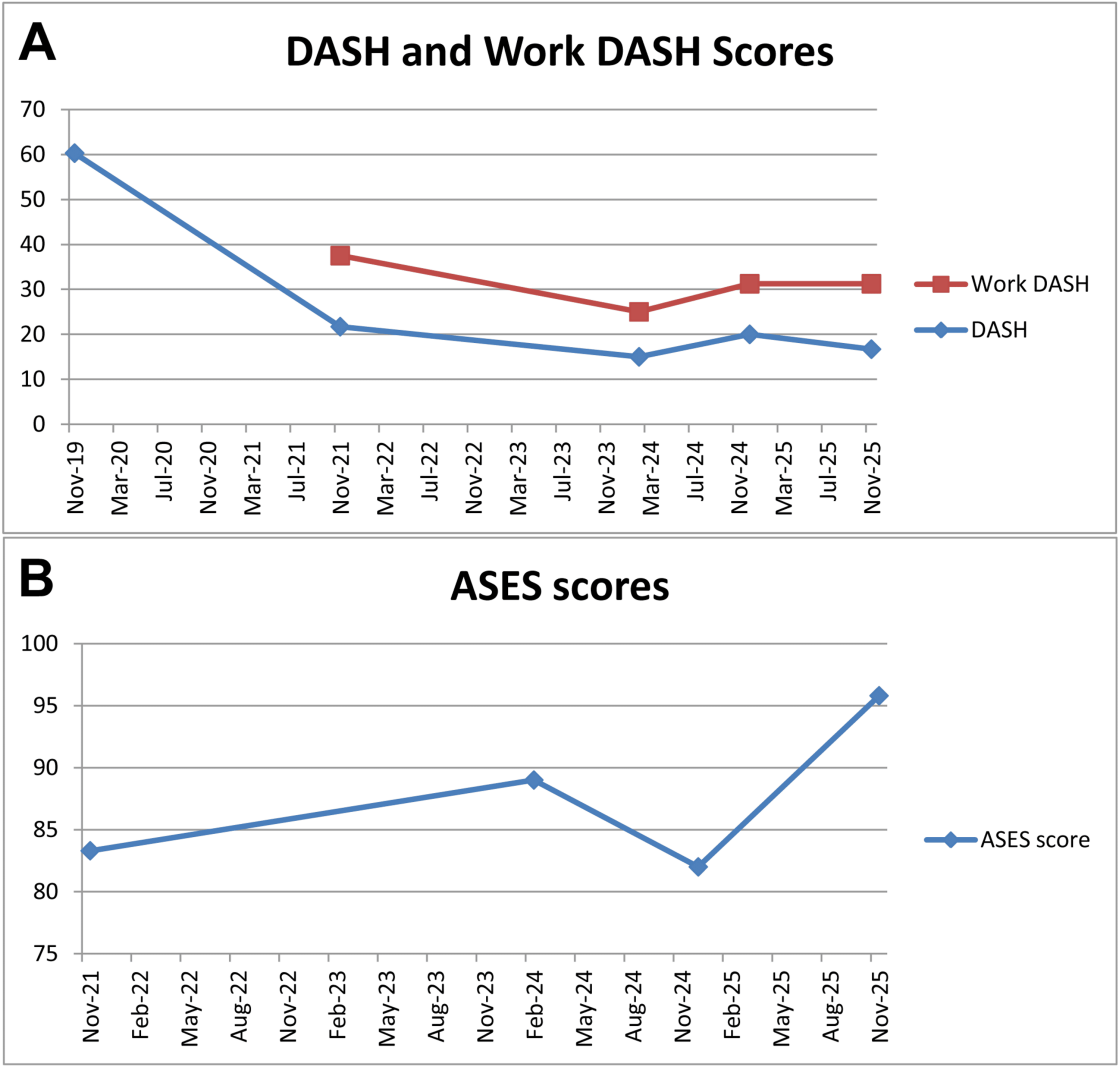
DASH and ASES scores. (A) DASH (total and “work”) scores, where 0 represents the highest/best level of function and 100 represents severe disability. The downward trends for the patient’s involved (left) upper extremity reflect improvement. (B) ASES scores (which focus on shoulder function), where 100 represents the highest/best level of function and scores above 80-90 are generally considered very good to excellent. Months and years are shown on the abscissa in (A) and (B). The upward trends for the patient’s involved (left) shoulder/arm reflect improvement. DASH: Disabilities of the Arm, Shoulder and Hand; ASES: American Shoulder and Elbow Surgeons.

The patient periodically completed a questionnaire that was customized for specific aspects of his activities of daily living, including prior and current work-related duties (Table 1). He also completed various validated outcome measures, including the Disabilities of the Arm, Shoulder and Hand (DASH), American Shoulder and Elbow Surgeons (ASES) score, SF-36, and the Single Assessment Numeric Evaluation (SANE) [53-58]. With the exception of the SANE values, the results of these outcome measures are summarized in Figure 6 and Table 2 (SF-36). At the 84-month follow-up visit, the patient reported an overall SANE value of 80 (100 is best/normal), with function reported separately for these regions: 100 for shoulder, 95 for arm, and 60 for hand. He stated that the general use and improvement of his left upper extremity was “good,” though he described his hand function as “fair” due to reduced dexterity and sensation (as reflected in the SANE and DASH scores). Notably, in early 2024 the patient had open-heart surgery with a valve replacement, which likely contributed to some reduction in his overall health values reported in Table 2.

**Table 1:** Time to improvement after final surgery for various activities/symptoms. Improvements reported by the patient, in months (mo), in functional status with respect to specific symptoms and work-related and other activities of daily living. Notably, the patient reported that some substantial improvements in specific tasks and symptoms did not occur until approximately 5-6 years after the final clavicle surgery. Such details are not reflected in the general subcategories of the SF-36 (Table 2). mo: Months; N/A: Patient did not reach the specified level of improvement; *: 60% improvement at 7 years; uses index finger and thumb to type ~35 words/minute.

Symptoms or Activities of Daily Living	Notable Improvement	Significant Improvement	100% Improvement
Hand feels like it is asleep	36 mo	72 mo	n/a
Hand always has a cold feeling	36 mo	72 mo	n/a
Forearm very sensitive/ache	16 mo	28 mo	32 mo
Arm ache	19 mo	31 mo	74-78 mo
Open door	23 mo	25 mo	74-78 mo
Lock door with key	24 mo	29 mo	74-78 mo
Button pants	19 mo	20 mo	60 mo
Button shirts	19 mo	23 mo	74-78 mo
Cut steak	23 mo	60 mo	74-78 mo
Thread a knot	23 mo	35-60 mo	74-78 mo
Hold a wrench	33 mo	66 mo	74-78 mo
Hold a grease line	24 mo	33 mo	74-78 mo
Type on keyboard	24-36 mo*	n/a	n/a
Use iPad touch screen	18 mo	27 mo	74-78 mo
Open jar	25 mo	60-72 mo	74-78 mo
Roll down window in truck	22 mo	31 mo	72 mo
Trim nails with nail clippers	24 mo	60-74 mo	74-78 mo
Zip up jacket	25 mo	25 mo	60 mo
Tie shoes	20 mo	23 mo	24 mo
Put on belt	23 mo	25 mo	26 mo
Open medicine bottle	23 mo	32-60 mo	70 mo
Put on deodorant	19 mo	23 mo	24 mo
Close door on vehicle	20 mo	22 mo	24 mo
Hold a bag or any weight	24 mo	24 mo	26 mo
Pick up small items	20 mo	60-72 mo	72-78 mo

**Table 2:** SF-36 results. *All questions are scored from 0 to 100, with 100 representing the highest level of function. Aggregate scores are compiled as a percentage of the total points using the RAND scoring method. It is notable that the patient’s general “physical functioning” (top row) did not improve after November 2021, which was three years after the final clavicle surgery. In contrast to these SF-36 values, Table 1 shows greater variation in the specific times (in months) to attainment of “significant improvements” in many of the patient’s specific activities of daily living. § In 2024, the patient had open-heart surgery with a valve replacement; this likely contributed to the lower scores in physical functioning and general health. † The “pain” was neuropathic (i.e., “burning” in quality) and was treated chronically with Neurontin. SF-36: 36-Item Short Form Health Survey.

Domain	Results (November 2021), 3 years after final clavicle surgery	Results (February 2024), 5.25 years after final clavicle surgery	Results (December 2024), 6 years after final clavicle surgery	Results (November 2025), 7 years after final clavicle surgery
Physical functioning	95	95	90	90§
Role limitations due to physical health	75	100	100	100
Role limitations due to emotional problems	100	100	100	100
Energy/Fatigue	75	85	80	85
Emotional well-being	92	92	92	92
Social functioning	87.5	100	100	100
Pain	67.5	80	67.5	67.5†
General health	85	80	85	75§

## Discussion

Our patient presented with a hypertrophic midshaft clavicle fracture nonunion that had occurred 15 years prior, and no surgery had been performed. Twelve days after our anatomic reconstruction, the patient noted diffuse numbness and weakness in his ipsilateral hand. This was considered post-surgical neurogenic TOS, and a final clavicle (revision) surgery aimed at increasing the space inferior and posterior to the clavicle unexpectedly worsened the patient’s deficits. It was then concluded that these two surgeries precipitated severe brachial plexopathy/PTS, and no further clavicle or brachial plexus surgery was performed. Given the prevalence of the term “Parsonage-Turner syndrome” (PTS) in the literature and our patient’s constellation of symptoms and delayed presentation (which is common in PTS) [59-61], we refer to his condition as “brachial plexopathy/PTS” in this report. This terminology reflects our initial interpretation of the most likely diagnosis in this case.

The main reason we believed that our patient had developed brachial plexopathy/PTS after his index left clavicle surgery was the 12-day delay in the manifestation of symptoms. This is also supported by Iyer VG et al.’s study of the clinical spectrum of post-surgery brachial plexopathy/PTS in patients referred to their electrodiagnostic laboratory. In their cohort of 202 patients, they differentiated between idiopathic PTS, definite post-surgical PTS, and probable post-surgical PTS. They excluded patients in whom positional pressure or perioperative nerve injury was more likely. Procedures that were anatomically remote from the brachial plexus were classified as definite post-surgical PTS, whereas procedures performed in the vicinity of the brachial plexus (cervical spine and shoulder) or in the ipsilateral upper extremity were classified as probable post-surgical PTS [21].

In this context, our patient’s case seemed to be consistent with “probable postoperative PTS.” In the 35 patients (17%) that Iyer VG et al. identified as having probable post-surgical “brachial plexitis” (herein we use “brachial plexopathy”), 17 (49%) had shoulder surgery before the onset of their brachial plexitis/PTS (but none were performed in the region of the clavicle or brachial plexus). In the 26 patients that they identified as having definite post-surgical brachial plexitis/PTS, only three of the surgeries were in the upper extremity (two surgeries involving the hand or wrist and one surgery involving the shoulder). Notably, the mean duration between surgery and symptom onset for all cases of post-surgical brachial plexitis/PTS (definite and probable) was two weeks (range: 3-28 days).

As described above, we later became aware of a published case report [37] that caused us to consider that: (1) a traction/stretch injury to the brachial plexus might better explain our patient’s symptoms in comparison to the initial diagnosis of PTS, and (2) the steps taken to decompress the brachial plexus likely exacerbated the traction injury and subsequent perineural edema/ischemia (similar to a “double-hit” phenomenon [37]) rather than further inciting an inflammatory/immune-mediated process. If this interpretation is correct, our patient may have been particularly vulnerable to a brachial plexus traction injury due to adaptive shortening and the presence of fibrous adhesions between the peribrachial plexus tissue and the chronically shortened clavicle, consistent with several specific cases later identified in our literature search (Table 3) [1,24,25]. We also later became aware of additional reports of patients who had circumstances and symptoms associated with management of a clavicle fracture malunion that have some resemblance to those in our patient’s case [5,10,25,27,37,62-67]. This led us to ultimately conclude that acute brachial plexopathy/PTS might not have been the correct diagnosis. Our literature search for reports of brachial plexopathy/PTS after reconstruction surgery for a clavicle fracture nonunion suggested that it would be an extremely rare occurrence in this setting; in fact, we could not locate any cases where this diagnosis was associated with any type of clavicle fracture or surgery for a clavicle fracture, whether acute, sub-acute, or chronic (i.e., nonunion). We offer compelling opposing arguments below that support explanations that our patient’s symptoms were either most consistent with brachial plexopathy/PTS or, more likely, iatrogenic postoperative traction (lateral stretch injury) axonotmesis. However, because no definitive intraoperative or histopathological confirmation was available, our conclusion remains inferential.

**Table 3:** Cases of iatrogenic brachial plexus traction/stretch (clavicle lengthening) and compression injuries following clavicle surgery. *: Lateral stretch injury due to clavicle lengthening; §: More direct compression injury; ‡: ORIF ORIF: Open reduction and internal fixation; PTS: Parsonage-Turner syndrome; ROM: Range of motion; -----: Data not provided; F: Female; M: Male; TOS = Thoracic Outlet Syndrome; rhBMP-2 (or Rh BMP-2): Recombinant human Bone Morphogenetic Protein-2; NCS: Nerve Conduction Study.

Author	Patient age (years), sex	Injury and initial surgery	Symptoms	Suggested mechanism of brachial plexopathy	Treatment	Diagnosis	Duration of follow-up	Time from fracture to surgery	Outcome
*Ring D, Holovacs T (2005) [62]	69, F	Midshaft clavicle fracture; intramedullary fixation	Weakness of elbow flexion and wrist extension; loss of shoulder flexion, abduction, and external rotation	Brachial plexus palsy likely resulted from manipulation of fracture fragments for medullary canal reaming	Consultation with a surgeon experienced in brachial plexus injuries; observation; no further surgeries; wrist splint	Upper trunk palsy, not PTS	6 months	~4 weeks	Complete recovery
	36, M	Midshaft clavicle fracture; intramedullary fixation	Upper limb: no motor or sensory function; skin mottled and slightly cool	-----	-----	Complete brachial plexus palsy, not PTS	6 months	~4 weeks	Complete recovery
	30, F	Midshaft clavicle fracture; intramedullary fixation	Global shoulder weakness; weakness of elbow flexion and extension, and wrist and digit extension	-----	-----	Upper trunk palsy, not PTS	6 months	~1 week	Complete recovery
§Thavarajah D, Scadden J (2012) [5]	43, F	Midshaft clavicle nonunion; ORIF with autograft from hypertrophic callus	0/5 strength and absent two-point discrimination in the majority of the upper extremity; 3/5 wrist extension; two-point discrimination present in superficial radial nerve distribution	Pressure on the plexus due to failure to remove hypertrophic tissue surrounding the fracture site	Plexus exploration with removal of callus and plate, plexus neurolysis, and excision of the middle third of the clavicle	Incomplete plexus palsy with sparing of the posterior cord, not PTS	-----	8 months	Near-complete recovery of motor and sensory function
§Namdari S et al. (2012) [63]	39, F	Widely displaced midshaft clavicle fracture nonunion; ORIF and iliac crest graft	Elbow extension 3/5, wrist extension 4/5, wrist flexion 1/5, hand function 1/5; decreased sensation in distal radial, ulnar, and median nerve distributions	Reduction of the nonunion caused a posteroinferior butterfly fragment to compress the plexus	Surgical excision of the butterfly fragment; plate left intact	Diffuse plexopathy affecting the lower trunk, not PTS	4 months	~7 months	Moderate improvement in sensation and strength; persistent reduced pinprick sensation in the dorsal-radial aspect of the hand and forearm
*Rosati M et al. (2013) [65]	48, F	Middle-third clavicle fracture; ORIF	Numbness and tingling in ulnar and medial nerve distributions, with later radial nerve deficit, including weakness of the extensor carpi ulnaris and interossei (3/5)	Narrowing/irritation of neurovascular tissues beneath the clavicle	Mobilization of fracture stumps to “widen” the costoclavicular space suggested; however, review of radiographs suggests the clavicle was allowed to shorten* after hardware removal	Direct compression leading to TOS suggested; however, review of the postoperative radiograph after hardware removal suggests clavicle lengthening* as the cause, not PTS	~5 months	~4 months	Near-complete recovery; cross-finger test normal; paresthesias and hyperhidrosis resolved
*Gross CE et al. (2013) [37]	78, F	Acute midshaft clavicle fracture; ORIF performed 5 weeks after injury	24 hours after surgery: deltoid 2/5, wrist and digital extensors 0/5, triceps 3/5, biceps 3/5	Acute lengthening/stretch of the plexus after adaptation to a shortened clavicle for 5 weeks	Hardware removal to allow the clavicle/plexus to return to the preoperative shortened length	Acute brachial plexopathy, not PTS	8 months	5 weeks	Complete recovery at 8 months; clavicle nonunion
*Jeyaseelan L et al. (2013) [25]	24, M	Acute midshaft clavicle fracture; ORIF	C5/C6 palsy; deltoid 4/5, supraspinatus and infraspinatus 0/5; shooting arm pain	Tethering of the nerve to the undersurface of the clavicle by scar tissue at the fracture site	Brachial plexus exploration and neurolysis of the suprascapular nerve	C5/C6 palsy, not PTS	12 months	24 days	Near-complete recovery of weakness after 12 months
	Cohort: 19 males, 2 females; mean age 36	3 middle-third, 14 midshaft, and 4 lateral-third fractures; all underwent fixation	Weakness in all; plexus palsy (14%); C5/C6 complete or partial palsy (76%); C5/C6/C7 complete palsy (10%); reduced sensation (90%); pins and needles (57%); pain (76%)	Plexus tethering within scar tissue causing displacement (95%); in one patient, tethering to two prominent screws	Exploration in all; 86% supraclavicular only; 14% both infraclavicular and supraclavicular; neurolysis in all	Complete plexus palsy (3); C5/C6 complete (10); C5/C6 partial (6); C5/C6/C7 palsy (2), not PTS	12 months	Mean 19 days (range 4-31 days)	Sensation and weakness improved postoperatively in all patients
*Matthews JR et al. (2015) [68]	68, M	Midshaft clavicle nonunion; ORIF	Weakness and paresthesias in median and ulnar distributions; paresthesias in radial distribution	Although recombinant human bone morphogenetic protein-2 (rhBMP-2) was suggested, clavicle lengthening appears more likely (as also concluded by Larrota et al., (2024) [67]	Observation; nerve conduction studies; no revision surgery	Brachial plexopathy, possibly complicated by diabetic peripheral neuropathy, not PTS	24 months	3 months	Symptom-free with normal motor and sensory exam; self-reported satisfactory outcome
*Johnson CS et al. (2020) [64]	54, M	Midshaft clavicle nonunion; ORIF with iliac crest graft	Paresthesia; arm numbness distal to the deltoid; progressive weakness beginning with the biceps; decreased pain sensation in ulnar distribution; wrist extension 2/5	Clavicle lengthening caused stretch injury of the plexus	Close monitoring and occupational/physical therapy; no revision surgery	Brachial plexopathy, not PTS	12 months	5 months	Strength and sensation returned to normal, aside from occasional neuralgia and some limited forward ROM
Kim Y et al., (2020) [28]	56, M	Midshaft clavicle nonunion with prior surgery; radiographs showed a loosened implant; revision ORIF caused lengthening, with a bone fragment also compressing the subclavicular space	Numbness and decreased strength; 1/5 thumb extension/flexion, wrist extension, and finger extension; 3/5 wrist flexion and elbow flexion/extension	Compression due to a fibrotic mass surrounding a small bony fragment inferior to the clavicle	Surgery to remove the implant and create superior angulation to decompress the brachial plexus	Brachial plexus neuropathy	6 months	Unknown (first operation 5 months prior)	Shoulder abduction and flexion 3/5 and 4/5, respectively; hand and elbow function fully recovered
*Cao Z et al. (2021) [68]	34, F	Clavicle fracture; ORIF	Weakness in upper arm/forearm; reduced ROM in elbow, wrist, and fingers; deficits in wrist extension, thumb extension, and elbow flexion; strength 2/5 in deltoid, biceps, and extensor pollicis longus	Multiple theories: possible puncture; sharp fracture ends scratching nerves during reduction; heat-related injury from electrocautery	Oral methylcobalamin and electrical stimulation; no revision surgery	Brachial plexopathy	2 months	Immediate	Motor function, ROM, and strength fully restored; no sensory abnormality compared with contralateral side
§McGillivray MK et al. (2022) [27]	35, M	Clavicle nonunion; revision ORIF with iliac crest graft	Forearm/hand numbness; weakness in elbow flexion, wrist flexion/extension, finger flexion/extension, and intrinsic hand muscles	Posterior/deep callus compressed the plexus during reduction	Implant removal and plexus decompression (within 16 hours); two days later plexus and subclavian vessel exploration, callus removal, implant revision, and bone grafting	Brachial plexopathy, not PTS	10 months	-----	Near-complete recovery at 10 months with 4+/5 deltoid strength; pronation improving
	45, F	Clavicle nonunion; ORIF complicated by infection; second ORIF with iliac crest bone graft one year later	Paresthesias distal to the elbow; progressive weakness affecting medial-cord-innervated musculature	Clavicle reduction caused callus to compress the medial cord against the first rib	Brachial plexus and subclavian vessel exploration; callus excision; clavicular implant revision; bone graft	Brachial plexopathy, not PTS	-----	Not specified (likely delayed fixation)	Resolution of paresthesias/neuropathic symptoms; residual medial-cord-innervated weakness
*Larrota G et al. (2024) [67]	28, M	Midshaft clavicle fracture nonunion; ORIF and bone graft application	Neuropathic pain and weakness; reduced touch sensation	Infraclavicular brachial plexus lesion involving posterior cord and musculocutaneous nerve	Conservative management with neuromodulator medications, observation, and physical therapy	Brachial plexopathy, not PTS	12 months	5 months	Strength and sensation fully restored
§Shin D, Han JH (2025) [29]	61, F	Midshaft clavicle fracture; ORIF with bone grafting and excision of deformed bone at prior fracture site	Decreased sensation in ulnar and median distributions; motor deficits 1/5 to 3/5 in elbow, wrist, fingers, and thumb	MRI/CT suggested localized compression by clavicle fragments	Revision surgery to remove plate and excise butterfly fragment beneath the clavicle	Secondary TOS due to indirect nerve compression by reduced clavicle fragments, not PTS	12 months	2 weeks	Significant motor/sensory recovery; partial subjective sensory deficits remained
*Current case	56, M	15-year midshaft clavicle nonunion; ORIF followed by revision surgery to decompress the subclavicular region	Diffuse ipsilateral hand numbness and weak wrist extension, finger flexion, and grip strength; all worsened after revision surgery	Plexus tethered by scar tissue on a chronically shortened clavicle; reduction imparted lateral stretch (traction injury)	Referred to brachial plexus specialist; Oberlin nerve transfer, cubital tunnel release, and carpal tunnel release; no plexus neurolysis	Initially brachial plexopathy/PTS; later concluded traction-induced axonotmesis	7 years	15 years	Good overall recovery (fair hand function); by 6 years, recovery plateaued with persistent hand numbness and occasional cramping

Lengthening/stretch injuries of the brachial plexus associated with surgery for clavicle fracture nonunions and delayed fixation

Our literature review revealed reports of patients with brachial plexus deficits after ipsilateral surgery for clavicle fracture nonunions or delayed fixation that resemble some of our patient’s symptoms (Table 3). However, the deficits in these patients were considered traction-related (lateral stretch) or direct compression-related neurapraxia/axonotmesis injuries, and none were considered to be brachial plexopathy/PTS. Many of these patients recovered within a few weeks to months after the aggravating factor (bone fragment, scar tissue, hardware, bone alignment, or acute clavicle lengthening) was removed or rectified. In some cases, the symptoms resolved without the need for surgery [62,64,67,68,69]. Five representative reports are summarized below:

Ring D and Holovacs T [62] describe three patients (69, 36, and 30 years old) with brachial plexus palsies resulting from intramedullary fixation of midshaft clavicle fractures. Two of these patients underwent delayed fixation (three to four weeks after injury) of displaced and shortened clavicular fractures. The brachial plexopathy was recognized in the first patient the day after surgery, and it is implied that the other two patients also had acute manifestations. Without further operative intervention, all patients exhibited neurological improvement within two weeks and complete recovery within six months. They believed that injury to the brachial plexus likely occurred with delivery of the fracture ends from the wound to facilitate intramedullary reaming, an operative step not taken in our patient or in the patient described by Gross CE et al. [37].

Thavarajah D and Scadden J [5] describe a 43-year-old patient with a hypertrophic nonunion of a midshaft clavicle fracture. At eight months after the original injury, they performed ORIF with a dorsal metal plate and screws. Immediately after surgery, impaired neurological function was detected, reflecting an incomplete plexus injury. After an unspecified interval, the patient underwent a second surgery to remove the hardware and the middle one-third of the clavicle and to perform neurolysis of the brachial plexus. Postoperatively, the patient’s symptoms notably improved, with increased power from C5-T1 and a corresponding return of two-point discrimination.

As noted above, Gross CE et al. [37] describe a 78-year-old who developed brachial plexopathy 24 hours after ORIF of a delayed union clavicle fracture (surgery was done after five weeks of conservative management). Symptoms improved after the plate was removed and the clavicle was allowed to shorten to the pre-operative length. They speculated that after injury, due to fragmentation and overall shortening of the clavicle, the cords of the brachial plexus had become contracted due to the decreased infraclavicular space. They also speculated that inadequate soft tissue release during the initial surgical dissection may have contributed (as recommended in a subsequent report by Jeyaseelan L et al. [25]). They suggested that the delay in presentation was due to a “second-hit” phenomenon. The neurapraxia due to acute clavicle lengthening was the “first hit” (i.e., a predominantly mechanical perturbation), and subsequent postoperative edema may have caused a second hit (i.e., a predominantly physiological perturbation). They speculated that the two hits combined to cause delayed neurological dysfunction starting at 24 hours postoperatively.

Jeyaseelan L et al. [25] describe 21 patients who had brachial plexus injuries as a direct consequence of delayed fixation (mean = 19 days, range 4-31 days) of clavicle fractures. In their cohort, neurological symptoms developed immediately following fracture fixation. All of these patients underwent exploration; three (14%) underwent supra- and infraclavicular exploration and 18 (86%) underwent supraclavicular exploration only. The hardware in all cases was retained and no clavicle excision was done. Neurolysis was performed in all cases because the plexus was found to be tethered to the clavicle by scar tissue; the plexopathy improved after lysis of scar/adhesions. They recommend that in cases of delayed fixation, all adherent soft tissues must be thoroughly released from the clavicle before the fragments are mobilized and that no shortening of the clavicle occurs during fixation.

Johnson CS et al. [64] describe a 54-year-old with an atrophic midshaft clavicle fracture nonunion treated with plate fixation and iliac crest bone graft five months post-injury. Paresthesia was noted the evening of surgery, progressing to arm numbness distal to the deltoid, along with increasing weakness of his arm and forearm muscles. CT imaging showed proper clavicle alignment without hardware complication or hematoma. By postoperative day seven, the patient’s numbness improved, but he began experiencing electrical sensations radiating distally along the posterior aspect of his arm. Wrist extension improved from 2/5 to 4/5. The patient participated in occupational/physical therapy (revision surgery was not done). His strength and sensation had returned to near normal by six months, though he did report occasional neuralgia and limited forward range of motion. At one year postoperatively, his status was unchanged. Similar to Gross CE et al. [37], the authors attributed the complication to acute lengthening of the clavicle, causing an iatrogenic stretch injury of the brachial plexus. However, unlike Gross CE et al., their patient was able to achieve near-complete recovery without further surgery, likely due to a lower magnitude of lengthening (5.9 mm vs. 27 mm).

The possibility that hardware removal and partial clavicle excision would have hastened our patient’s recovery

We believe our patient’s recovery would have been substantially hastened if the hardware had been removed in a timely manner, allowing the clavicle to shorten back to baseline, as done by Gross CE et al. [37,65,66], and/or by excising the middle portion of the clavicle [5]. However, at that time we were also highly concerned about perioperative mortality and morbidity from a subclavian vessel injury [30,31,70], particularly if subclavicular dissection had become necessary [25]. In view of our patient’s improvement, though slow, it was unclear whether surgical intervention would change the natural history of what we believed to be brachial plexopathy/PTS [13,23]. Surgical intervention is rarely required for brachial plexopathy/PTS; however, neurolysis for hourglass constrictions of peripheral nerves (not seen in our patient) may be considered after 12 months of failed conservative treatment [23].

Concerns for significantly reduced shoulder function from partial clavicle excision

When considering options for immediate revision surgery in our patient (i.e., hardware removal, partial clavicle excision, and brachial plexus neurolysis), we were also concerned that excision of a large segment of the clavicle might result in dysfunction that would make a return to manual labor unlikely. Total and partial claviculectomies have been reported to yield good, but not symptom-free, results [30,71,72]. For example, Green RM et al. [71] describe the shoulder function of 11 patients who underwent claviculectomy. Three of these patients performed manual labor at work and reported suboptimal outcomes: (1) a 25-year-old automobile mechanic reported “shoulder pain with repetitive pulling movements” and “occasional ulnar nerve distribution pain”; (2) a 32-year-old diesel mechanic reported that his “shoulder aches with repetitive use”; and (3) a 38-year-old electrician reported that he “cannot do overhead drilling,” and his shoulder “clicks and arm gets numb when he sleeps on the ipsilateral side.” Options for patients with unsatisfactory outcomes after total claviculectomy include reconstruction with an allograft or a vascularized fibula graft [73,74].

How long after the onset of non-PTS brachial plexopathy after clavicle surgery is surgical intervention no longer helpful?

A literature review by Martin E et al. [75] on traumatic brachial plexus injuries (not PTS) provides insight into when it might be too late to decompress around these nerves. Across 43 studies, most showed significantly better motor outcomes when surgery was performed within six months, with some citing shorter optimal windows. Pain and quality-of-life scores were also significantly improved with shorter delays. Among the 569 patients with individual-level data, 66% underwent surgery within six months and 27% within three months. The highest percentage achieving a strength grading of 3 (90%) occurred in the group operated on within three months. The percentages decreased with longer delays (36% achieving grade 3 strength with delays >12 months). The median time to surgery among patients recovering to >3 strength was four months versus seven months for those reaching ≤3.

Even if our patient had brachial plexopathy/PTS, there is little concern that surgical hardware removal to allow clavicle shortening would have been deleterious (assuming no conventional postoperative complications occurred). In other words, such surgical intervention could have been done regardless of the underlying etiology because, although this might not have been helpful for brachial plexopathy/PTS, it would not be expected to cause additional deficits. However, in patients with the hereditary form of brachial plexopathy/PTS, additional surgery may induce recurrent attacks [76].

Availability of a surgeon comfortable with performing brachial plexus neurolysis

An additional complicating factor in our decision-making process was the lack of availability of a surgeon comfortable performing dissection/neurolysis of the brachial plexus during or soon after surgery. We advise orthopaedic surgeons who are considering performing reconstruction surgery for a clavicle fracture nonunion (especially if very chronic) to ensure access to surgeons skilled in brachial plexus dissection and vessel injury management during surgery or in the perioperative period. Additionally, intraoperative neuromonitoring can be useful for minimizing the risk of additional brachial plexus injury and determining the adequacy of neural decompression during revision surgery for clavicle fractures [77].

Highly variable recovery of non-PTS and PTS brachial plexopathy

The timeline of recovery from non-PTS brachial plexopathies after clavicle fractures (with or without surgical intervention) is highly variable (Table 3). Similarly, in cases of brachial plexopathy/PTS (again, no cases have been reported in association with clavicle fractures, with or without surgery), the duration of recovery is also highly variable, with pain usually resolving in a few days to weeks but weakness generally resolving over six months to three years [18,21,45]. For example, in a study of 99 patients with brachial plexopathy/PTS, Tsairis P et al. [45] found that 36% of patients had complete functional recovery within one year, 75% by two years, and 89% by three years. Patients with upper trunk injuries had the most optimal outcomes, with near-complete functional recovery by one year. In contrast, patients with lower- and middle-trunk (hand and forearm) involvement (such as our patient) typically did not normalize until 1.5 to 3 years. However, 18 of their patients had more prolonged courses of recovery and were followed for five years, with two having residual minimal clinical findings. Recent work from the Netherlands [19,41] also shows that many patients with brachial plexopathy/PTS had lingering impairments in strength, pain, fatigue, psychological distress, functional status, and quality-of-life questionnaires at 2.5-year follow-up. Geertzen JH et al. [42] also reported less-than-favorable long-term impairments in their 16 patients with brachial plexopathy/PTS who had a mean follow-up of eight years (range: 1-15 years). Of their 13 patients who were followed for at least eight years, many reported ongoing impairment: seven with pain, 11 with muscle weakness, seven with muscle atrophy, and five with paresthesias. Because of persistent impairments, all patients had to discontinue their sport activities, four received full disability payments, one took early retirement, and four switched from manual labor to office-based work (as done by our patient).

Double crush/triple crush possibility vs. the double-hit idea of Gross CE et al.

The double (or triple/multiple) crush hypothesis is often considered when peripheral nerve injuries/deficits are worse than expected. This consideration is based on the idea that compression or damage to a peripheral nerve proximally can predispose the nerve to additional damage distally [78-81]. The “double crush” phenomenon was first described by Upton AR and McComas AJ [82] in their study of the association between cervical root lesions in most (70%) of their 115 patients who had carpal tunnel syndrome or ulnar neuropathy at the elbow. However, this hypothesis has been questioned and may be overused [83]. If double crush syndrome is valid, then it is a consideration in our patient’s case due to his ipsilateral cervical disc bulge and contralateral brain lesion, both of which may have predisposed him to enhanced distal nerve impairment after his clavicle surgeries. Although the CNS component is not classically considered in the context of double crush syndrome, it might contribute because it is hypothesized that lesions of the CNS might lead to “central sensitization,” which is a state of CNS hyperexcitability [84]. In our patient’s case, it is also theoretically possible that a triple/multiple-crush phenomenon (attributed to a systemic/metabolic factor) was at play in addition to the compression-type factors characteristic of double-crush syndromes. The only systemic/metabolic factor that might have been at play in this context was his type 2 diabetes [85,86].

The double crush phenomenon differs from the “double-hit” phenomenon described by Gross CE et al. [37]. In that paper, they did not reference any studies or case reports that used this phrase or addressed its prevalence or etiology. We also found no mention of this phenomenon in any of the publications in our literature search (including those listed in the literature review by Borole A et al. [1]) of neurovascular complications following clavicle fracture fixation. Double-/second-hit phenomena that are more consistent with that described by Gross CE et al. have been described in the context of orthopaedic trauma surgery [87,88]. Nevertheless, we believe that this mechanism is a highly plausible explanation for the postoperative signs and symptoms exhibited by our patient.

## Conclusions

The 12-day delay before the onset of weakness and numbness in our patient seemed to support an inflammatory or immune-mediated etiology. This led us away from surgical intervention. In retrospect, we speculate that revision surgery, including clavicle shortening and brachial plexus neurolysis, might have reduced the duration of our patient’s recovery and improved his outcome, thus suggesting a non-PTS etiology. For patients who cannot, or elect not to, undergo additional surgery to shorten the clavicle under similar circumstances, our case illustrates that fair-to-good functional recovery of the limb is still achievable, particularly if the involved hand is non-dominant. However, this process may take several years, and targeted interventions such as a modified Oberlin nerve transfer, carpal and cubital tunnel decompression, and extensive hand therapy can significantly enhance long-term outcomes.
